# Refractory antimelanoma differentiation-associated gene 5 antibody negative ulcerative dermatomyositis responsive to mycophenolate mofetil

**DOI:** 10.1016/j.jdcr.2024.05.017

**Published:** 2024-05-21

**Authors:** Sumeyye Ozer, Banu Farabi, Rebecca Kann, Mehmet Fatih Atak, Kenneth Shulman, Shoshana Marmon

**Affiliations:** a Rao Dermatology, New York, New York; b New York Medical College, Valhalla, New York; c NYC Health + Hospitals/Metropolitan, New York, New York; d NYC Health + Hospitals/South Brooklyn Health, Brooklyn, New York; e Dermpath Diagnostics, Westchester, New York

**Keywords:** anti-MDA-5 negative dermatomyositis, connective tissue disease, interstitial lung disease, treatment resistant dermatomyositis, ulcers

## Introduction

Dermatomyositis (DM) is an autoimmune disorder characterized by various clinical presentations associated with distinct immunologic profiles.^1^ The presence of ulceration, is an infrequent cutaneous manifestation in adult DM and has mostly been reported in Juvenile DM or in adults with the melanoma differentiation-associated gene 5 (MDA-5) antibody positive subtype of DM, which is highly correlated with rapidly progressive interstitial lung disease (ILD).^2^^,^^3^ Herein, we present a case of DM in a Hispanic patient with multiple purulent ulcerative nodules and draining abscesses, who was negative for anti-MDA-5 and anti-MI-2 antibodies on serology and had no evidence of lung disease on imaging. Notably, immunologic profiles and overall clinical presentations of DM are less well-characterized in patients with skin of color with a particular lack of data among Hispanic patients.

## Case report

**Fig 1:**
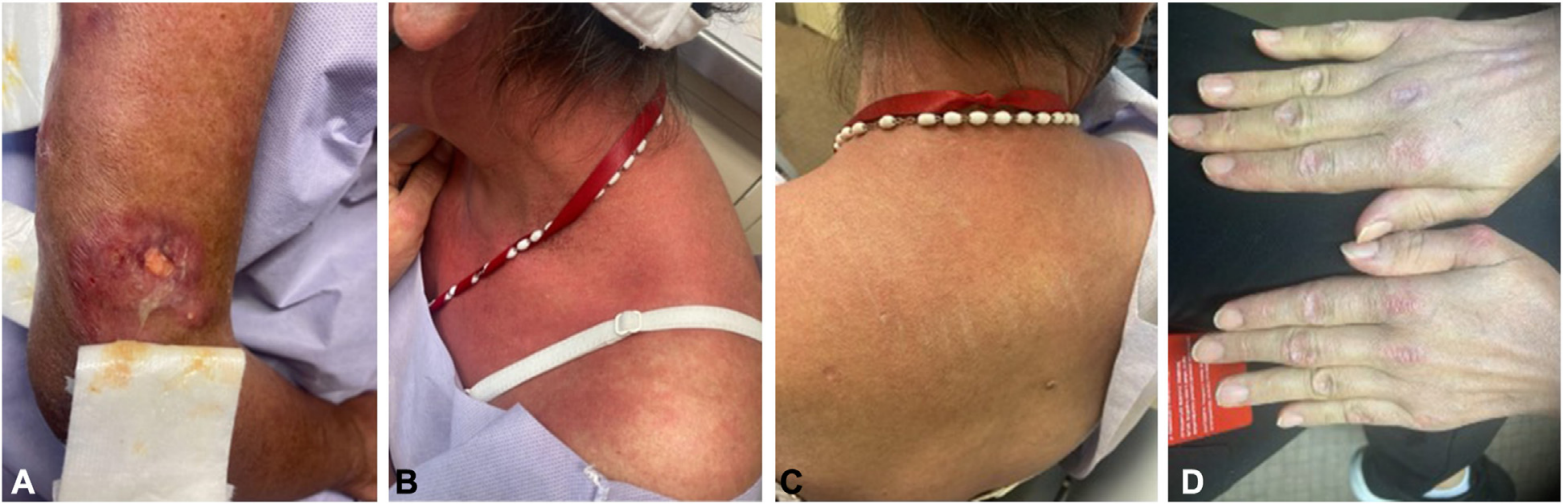
**A,** Purulent ulcerative nodules on patient’s upper extremity. **B, C,** Shawl sign consisting of flat, reddened area on the chest and back of the patient. **D,** Multiple erythematous papules on the proximal interphalangeals and distal interphalangeals.

A 52-year-old Hispanic woman with DM was referred to our dermatology department due to multiple large sores. The patient had presented to rheumatology 1.5 years earlier and was being treated with hydroxychloroquine and prednisone in addition to azathioprine and methotrexate. On examination, numerous purulent ulcerative nodules were identified on her trunk and extremities along with Gottron papules, shawl sign, facial erythema, and psoriasiform dermatitis in the auricular area (Fig 1, *A*-*D*).

Serology was positive for antinuclear antibody (1:1280), anti-Ro52, anti-beta-2 glycoprotein but negative for anti-MDA-5 and anti-MI-2 antibodies. Serology was also negative for cardiolipin antibodies, TIF1-y, NXP-2, and anti-jo/Pl7/Pl12. Creatinine phosphokinase levels were elevated. Imaging from high resolution computed tomography showed no evidence of ILD or malignancy.

**Fig 2:**
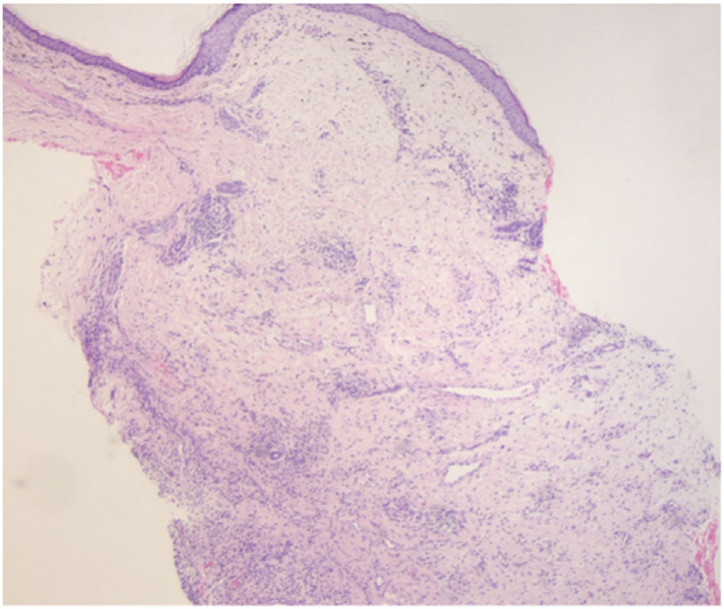
Punch biopsy from the edge of an ulcer revealed fibrosis and a focal mixed inflammatory infiltrate with lymphocytes, a few plasma cells and a few eosinophils. (Hematoxylin-eosin stain; original magnification: ×4.)

Tissue culture revealed growth of *Staphylococcus hominis*, an organism that is often part of the normal flora. This finding allowed us to rule out other infectious causes of ulcers and was suggestive of connective tissue disease as the underlying etiology. Histology from a punch biopsy of the edge of an ulcer revealed fibrosis and a focal mixed inflammatory infiltrate with lymphocytes, a few plasma cells and a few eosinophils (Fig 2). These findings were compatible with nonspecific chronic inflammation from a nearby ulcer and showed no evidence of epidermal interface change, granulomatous inflammation, vasculitis, perforating collagenosis, or necrobiosis lipoidica.

Despite strict sun protection and treatment with azathioprine, hydroxychloroquine, prednisone, high potency topical steroids, mupirocin, and repetitive courses of doxycycline and Keflex, the patient’s ulcers and muscle weakness persisted for over a year. At that point, azathioprine was replaced with 3000 mg of mycophenolate mofetil twice a day leading to the resolution of her ulcers and improvement of systemic symptoms.

## Discussion

In this case, we report a 53-year-old Hispanic woman with multiple large sores concurrent with muscle and joint pain and weakness. She presented with classic DM features, including shawl sign, Grotton papules, and muscle aches.^4^ However, our patient remained negative for anti-MDA-5 and anti-MI-2 antibodies.

Many patients carry myositis-specific antibodies that have been closely connected to specific disease phenotypes of idiopathic inflammatory myopathies. MI-2 antibodies have been associated with classic DM, its distinctive cutaneous features and muscle weakness. On the other hand, anti-MDA-5 positivity is classically described in clinically amyopathic dermatomyositis. Patients with this subtype of DM are reported to have skin ulcerations and ILD, but limited muscle involvement.^1^

Our patient’s immunologic and clinical profile does not match either of the aforementioned subtypes of DM. Ulcers in DM are mostly reported in juvenile type or anti-MDA-5 positive adult DM cases with presence of vasculitis in histopathology of the ulcer. There is only one case reported previously of an DM in an adult with ulcerative lesions but without vasculitic changes.^5^ This case stands out as a unique presentation of DM with nonvasculitic ulcerations in an adult.

We suspect that our patient’s clinical manifestations were not associated with the typical myositis-specific antibody profiles because DM can have varied presentations in different patient populations.

A few previously documented cases have reported the atypical presentation of DM in Hispanic patients. One case report discussed a 27-year-old Hispanic woman patient who had double anti-PL-7 and anti-MDA-5 positive amyopathic dermatomyositis with rapidly progressive ILD.^6^ Previously this rare presentation had only been reported in patients of Japanese descent. Another study identified novel cutaneous features; “*sunburn sign*” and “*suntan sign*” in Hispanic patients with DM.^7^ The sunburn sign appeared as bright red erythema on the forehead, cheeks, nose, and chin. The same areas subsequently developed brownish-gray hyperpigmentation with soft desquamation, referred to as the suntan sign. These studies, in conjunction with our case, emphasize that the clinical manifestations of DM may not consistently align with the serological markers traditionally associated with them. Furthermore, aspects such as race and ethnicity may have a potential impact on these clinical presentations, including the time it takes for symptoms to lead to diagnosis and subsequent treatment.^7^^,^^8^ This points to the necessity for a deeper understanding of the varied clinical and immunologic characteristics of DM in diverse patient groups.

## Conclusion

This case underscores that ulceration in a patient with DM can present in the absence of anti-MDA-5 antibody. It also highlights the refractory nature of ulcerative DM and the importance of employing different systemic medications to achieve control once an infectious etiology is ruled out. Finally, it emphasizes the importance of more comprehensive data regarding how DM manifests in diverse patient populations.

## Conflicts of interest

None disclosed.
